# Performance of radiomics-based artificial intelligence systems in the diagnosis and prediction of treatment response and survival in esophageal cancer: a systematic review and meta-analysis of diagnostic accuracy

**DOI:** 10.1093/dote/doad034

**Published:** 2023-05-26

**Authors:** Nainika Menon, Nadia Guidozzi, Swathikan Chidambaram, Sheraz Rehan Markar

**Affiliations:** Department of General Surgery, Oxford University Hospitals, Oxford, UK; Department of General Surgery, University of Witwatersrand, Johannesburg, South Africa; Academic Surgical Unit, Department of Surgery and Cancer, Imperial College London, St Mary’s Hospital, London, UK; Department of General Surgery, Oxford University Hospitals, Oxford, UK; Nuffield Department of Surgery, University of Oxford, Oxford, UK

**Keywords:** esophageal cancers, radiology, robotics

## Abstract

Radiomics can interpret radiological images with more detail and in less time compared to the human eye. Some challenges in managing esophageal cancer can be addressed by incorporating radiomics into image interpretation, treatment planning, and predicting response and survival. This systematic review and meta-analysis provides a summary of the evidence of radiomics in esophageal cancer. The systematic review was carried out using Pubmed, MEDLINE, and Ovid EMBASE databases—articles describing radiomics in esophageal cancer were included. A meta-analysis was also performed; 50 studies were included. For the assessment of treatment response using ^18^F-FDG PET/computed tomography (CT) scans, seven studies (443 patients) were included in the meta-analysis. The pooled sensitivity and specificity were 86.5% (81.1–90.6) and 87.1% (78.0–92.8). For the assessment of treatment response using CT scans, five studies (625 patients) were included in the meta-analysis, with a pooled sensitivity and specificity of 86.7% (81.4–90.7) and 76.1% (69.9–81.4). The remaining 37 studies formed the qualitative review, discussing radiomics in diagnosis, radiotherapy planning, and survival prediction. This review explores the wide-ranging possibilities of radiomics in esophageal cancer management. The sensitivities of ^18^F-FDG PET/CT scans and CT scans are comparable, but ^18^F-FDG PET/CT scans have improved specificity for AI-based prediction of treatment response. Models integrating clinical and radiomic features facilitate diagnosis and survival prediction. More research is required into comparing models and conducting large-scale studies to build a robust evidence base.

## INTRODUCTION

Outcomes in esophageal cancer are often related to inherent aggressive tumor biology but also due to challenges in early diagnosis and difficulties in assessing tumor response to an increasingly broader range of treatment modalities. Given the lack of non-invasive biomarkers, there is a significant reliance on radiological assessment at various stages of the cancer treatment pathway—diagnosis, radiotherapy planning, assessment of treatment response, surveillance, and prognostication. The interpretation of radiological images is limited by human factors, subjective visual interpretation and can often be time-consuming and inaccurate. With the advent of artificial intelligence (AI) in medical image interpretation, these limitations can potentially be overcome.

Within medicine, AI refers to the use of a system to replicate human cognition in the comprehension, analysis, and presentation of medical data. This can be achieved using machine learning, which is a specialized sub-field within AI that improves the performance of systems through repetition.^1^^, ^^2^ More recently, AI has been integrated into major imaging modalities to refine the way esophageal cancers are diagnosed and followed up. Machine learning models can be designed to train, validate, and test datasets for image interpretation. This is facilitated via the high-throughput extraction of large quantities of data from the images, known as radiomics.

Radiomics provide both a qualitative and quantitative perspective to the spatial relationship between pixels and signal intensities and additionally adds a layer of visual interpretation that may otherwise not be visible to the human eye.^3^^, ^^4^ This enables more consistent image interpretation with a focus on fine detail and patterns that may otherwise be missed. Within radiomics, textural analysis is the dominant area of interest in most research studies. Radiomics within imaging also offers the advantage of combining ML to obtain images; segment images into regions of interest (ROI) or volumes of interest (VOI); extract necessary details to construct a model; and validate the models. Although there are reviews that discuss radiomics in esophageal cancer, they are specific to cancer type or an imaging modality.^5–8^ This systematic review and meta-analysis consolidates the evidence for the role of radiomics throughout the esophageal cancer therapeutic pathway.

## METHODS

A systematic review of studies evaluating the use of AI for diagnostic and treatment purposes in esophageal cancer was performed. This systematic review follows the Preferred Reporting Items for Systematic Reviews and Meta-Analyses (PRISMA) guidelines.

### Search strategy and selection of studies

The search methodology was defined according to the PRISMA guidelines.^9^ A systematic literature search was carried out by two of the authors (NM and NG) using Pubmed, MEDLINE, and Ovid EMBASE databases (date range: 1992 to 6 January 2023) using the following search strategies with standard Boolean operators: ‘artificial intelligence’, ‘radiomics’, ‘machine learning’, ‘esophageal cancer’, and ‘oesophageal cancer’. Furthermore, the reference lists of included articles and review articles were hand searched for additional studies.

### Selection of studies: inclusion and exclusion criteria

Two authors (NM and NG) performed the literature search, and any disagreements were resolved by the senior author (SRM). Titles and abstracts were scanned, and irrelevant studies were excluded. Full text articles of the remaining studies were then retrieved and evaluated for inclusion. The inclusion criteria required articles to have reported on the use of radiomics in computed tomography (CT) and PET. English language studies for esophageal cancers of all types were included exploring the role of radiomics in the most commonly used imaging modalities such as positron emission tomography with 2-deoxy-2-[fluorine-18] fluoro-D-glucose integrated with computed tomography (^18^F-FDG PET/CT) scans and CT scan. A few studies reporting on the use of barium esophagograms were excluded as they were believed to be less commonly used as a key imaging modality in cancer diagnosis. Studies that discussed the use of AI in esophageal cancer but not specifically for radiomics were excluded. Comparative cohort studies, non-randomized prospective studies, and RCTs were included. However, case series, case reports, narrative reviews, editorials, and conference abstracts; studies without comparison groups; studies with pediatric patients or less than five patients; and publications in a non-English language were excluded.

### Outcome measures

The primary outcome measures are data related to the diagnostic accuracy of the imaging modality, including sensitivity, specificity, positive predictive value, and negative predictive value. We also collected other study data which included the year of publication, study design, sample size, country of study, type of patients, patient characteristics, outcome measures, and conclusions.

Data charting was performed by two authors (NM and NG), and cross-validated by a third author (SC). The data charting forms were formed through study group co-design. The forms were tested by the team to capture relevant study data. Data extraction was undertaken using a single charting and audit approach organized in a tabular form. The forms were then piloted on the five first studies to ensure the approach to data charting was consistent and in line with the research question and purpose. Based on these, we identified recurrent themes and data-points. Thereafter, a calibration exercise was performed on the next five studies. The results were discussed, and the data charting form was continuously updated in an iterative process to be inclusive for key features not initially listed.

### Statistical analysis

All statistical analyses were performed using STATA/SE, version 16.0 (StataCorp LLC, College Station, TX). The overall pooled estimate of sensitivity and specificity with their corresponding 95% confidence interval (95% CI) was calculated using the random-effects model by the metandi command in STATA/SE. Sensitivity was defined as the proportion of patients with esophageal cancer that were correctly confirmed by AI, while specificity was defined as correctly identifying patients without the disease. Forest plots were used to visualize the variation of the diagnostic parameters effect size estimates with 95% CI and weights from the included studies.

## RESULTS

**Fig. 1:**
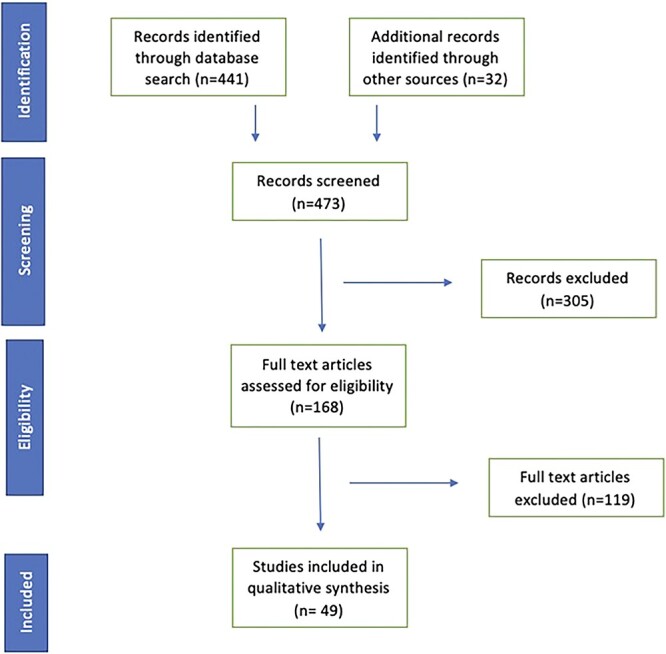
PRISMA flowchart.

Figure 1 describes the search methodology in detail. Fifty articles were included in this study, most of which were cohort studies (see Supplementary Tables for further detail). The results have been stratified into positron emission tomography with ^18^F-FDG PET/CT scan and CT scan sections.

### The use of radiomics in CT scans

#### Diagnosis

The diagnosis of esophageal cancer at an early stage remains a challenge and shapes clinical management for patients. Four studies have focused on identifying textural radiomics features and AI-based models to improve diagnostics.

Wang et al. in 2017 developed a support-vector machine (SVM) model to identify lymph node metastases and found this to be better than standard CT interpretation (area under the curve [AUC] 0.887 vs. 0.705).^10^ Similarly, Tan et al. studied 1576 radiomic features in the context of 230 esophageal squamous cell carcinoma (ESCC) patients on pre-treatment CT scans—these radiomic features were superior to size-based image features in predicting lymph node metastasis (AUC 0.758 training set, 0.773 test set).^11^ In 2021, Kawahara et al. used radiomics and machine learning to assess the degree of tumor differentiation from planning CT images of patients with locally advanced ESCC.^12^ Thirteen radiomic features were identified to assist in diagnosis between poorly differentiated tumors and moderately/well differentiated tumors. The AI system had an accuracy of 85.4% and specificity and sensitivity of 88.6% and 80.0%.^12^ A CNN-based model (VGG16) was developed based on 457 esophageal cancer patients and tested on 46 esophageal cancer patients (44 ESCC, 2 esophageal adenocarcinoma [EAC]) and was shown to have superior accuracy (0.842 vs. 0.836/0.808) and specificity (0.900 vs. 0.790/0.760) than two radiologists but a lower sensitivity (0.717 vs. 0.935/0.913).^13^

#### Treatment response

A total of 11 studies explored the role of radiomics in treatment response.

##### Models

Much like with ^18^F-FDG PET/CT scans, a variety of CT radiomics-based models have been developed to predict treatment response. Hu et al. used six peritumoral and seven intratumoral radiomics features from pretreatment CT scans of patients with ESCC undergoing chemoradiation and noted that the best performance was achieved by combining intratumoral and peritumoral features with an AUC of 0.852.^14^ Hu et al. then went on to compare handcrafted radiomics, deep learning algorithms/CNN-based, and clinical models to assess complete pathological response of ESCC to chemoradiation.^15^ Seven features were selected for radiomics and the model achieved an AUC of 0.725 in the validation cohort, this was lower than the CNN model (ResNET50) which used 14 features and achieved an AUC of 0.805.^15^ In support of the use of CNN models further, Li et al. performed a multicenter study to predict patient response to chemoradiation therapy.^16^ Using 3D-CNN the model achieved a PPV of 100% in the validation cohort.^16^ The model also used different radiotherapy regimens (large-field group and involved-field group) to predict treatment response and ultimately may be able to recommend individualized radiation treatment strategies on a per patient level.^16^ Furthermore, Jin et al. 2019 studied 94 patients and identified that a combination of radiomic and dosimetric features on CT produced a superior model at predicting treatment response than individual features.^17^ SVM and ANN models were used by Hou et al. to identify chemoradiation non-responders from responders in 49 patients using four principle methods; shape-based, histogram based, texture-based, and transform-based.^18^ Five radiomic features were identified that could successfully differentiate between chemoradiation responders and non-responders, these included Histogram2D_skewness, Histogram2D_kurtosis, GLSZM2D_LZE, Gabor2D_MSA-54, and Gabor2D_MSE-54.^18^ Yang et al. identified further radiomic features selected by LASSO in patients with ESCC with an AUC of 0.84–0.86 in the training cohort and 0.71–0.79 in the test cohort.^19^ Similarly, Riyahi et al. created an SVM-LASSO model with a Jacobian map with an AUC of 0.94, although the cohort itself was only 20 patients.^20^ Although Larue et al., similarly showed that radiomics models were superior to clinical models, Luo et al. demonstrated that a nomogram model that is a composite of radiomic features and clinical TNM staging was better.^21^^, ^^22^ Furthermore, the addition of ^18^F-FDG PET imaging to CT scanning (as demonstrated in the section above) was good at predicting treatment response and loco-regional disease control after neoadjuvant chemoradiotherapy.^23^

Pre-therapeutic CT scans have been used to predict the treatment response to immune-checkpoint inhibitors plus chemotherapy in 64 patients with advanced ESCC.^24^ Five features and support vector machine algorithms were used to build two-dimensional and three-dimensional radiomic models.^24^ The two-dimensional model outperformed the three-dimensional model in selecting which patients would benefit from this treatment strategy. The two-dimensional corrected model had an accuracy of 79.6% in the validation cohort.^24^

##### Pooled analysis

**Fig. 2:**
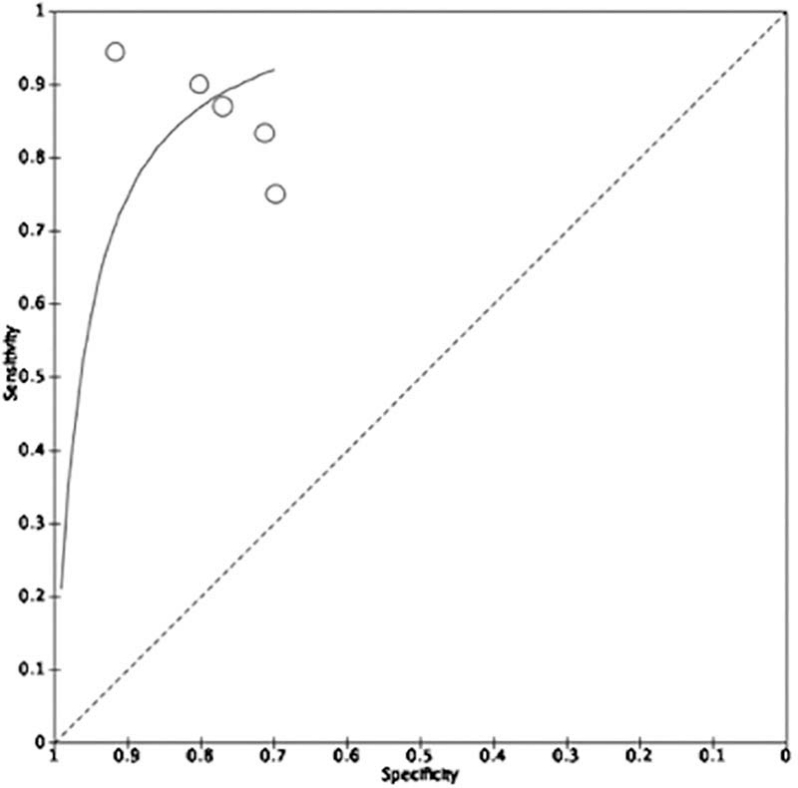
Treatment response in patients with esophageal cancer using radiomics in CT scans.

**Fig. 3:**
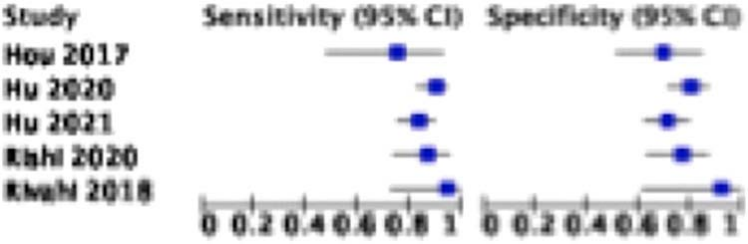
Treatment response in patients with esophageal cancer using radiomics in CT scans.

Five studies involving 625 patients provided sufficient data of true positive, true negative, false positive, and false negative rates for the calculation of sensitivity and specificity. All five studies assessed the treatment response in patients with esophageal cancer using CT scans. The pooled sensitivity and specificity were 86.7% (81.4–90.7) and 76.1% (69.9–81.4), respectively, as visualized on the forest plot and summary ROC curve (Figs 2 and 3). There was evidence for significant heterogeneity between studies (I^2^ = 64%).

#### Survival

Six studies focused on the use of radiomics in CT scans to predict survival in esophageal cancer. The reporting of the studies in this section meant that a meta-analysis could not be performed for this section, therefore, a narrative review has been provided instead.

Textural features such as zone distance variance GLDZM was significantly associated with overall survival. The development of AI-based CT and histopathological imaging models can be used to predict survival. A single center study consisting of 153 patients with ESCC developed a combined model integrating CT and histopathology interpretation, which showed an improved c-index (0.694) compared to individual CT or histopathology AI models alone.^25^ It was found that the features in histopathological images had a closer correlation to survival with more accurate prediction than CT alone.^25^ The algorithms were used successfully to predict 1–5 year mortality results with sensitivity and specificity of 78.1% and 84.7% at 1 year and 80.7% and 86.5% at 5 years, respectively.^40^ Interestingly, histopathological features were found to be more useful at predicting survival than radiomic features alone.^25^

Deep learning radiomics has followed on from handcrafted radiomics to further characterize tumor regions on CT scans in the prognostication of esophageal cancers after treatment. Handcrafted radiomics involves human-determined key tumor-related radiological features and regions of interest within the tumor, with AI introduced in the later stages to identify and consolidate these relevant features. More recently, deep learning radiomics integrates machine learning at an earlier stage with superior outcomes compared to handcrafted radiomics, by learning specific radiological features and eliminating the human aspect of image interpretation altogether.^26^ Much like Hu et al. noted for ^18^F-FDG PET/CT scans, Wang et al. also noted that deep learning models were better at survival prognostication than handcrafted or clinical models.^15^^, ^^26^ As shown in previous sections, composite models of clinical, including morphological characteristics such as wall thickness, and radiomic features were good at predicting survival.^27^^, ^^28^

#### Other (radiotherapy planning, incidental esophageal cancers)

The measured fields prior to radiotherapy are divided into gross tumor volume (main bulk of the tumor) and clinical target volume (surrounding subclinical malignant disease with likely microscopic tumor burden). Radiotherapy planning for patients with esophageal cancer is reliant on quantifying these boundaries on CT scans, a time-consuming and often difficult process with intra- and inter-user variability. This can be particularly challenging in the adjuvant setting with the primary tumor resection and additional postoperative changes visible on the CT images. Two studies explored the role of radiomics in radiotherapy planning. The use of AI in measuring the gross tumor volume and clinical target volume can standardize the process, with comparable results to clinicians, however, in much less time.^29^ The development of CNN in radiomics has enabled an automated process for organ segmentation in targeted radiotherapy built on deep learning and more recently, improved by a three-dimensional model.^30^ In the adjuvant setting, an AI model built for estimating clinical target volume averaged at 25 seconds per patient.^31^

Furthermore, Sui et al. studied the role of radiomics in identifying incidental, false negative esophageal cancers.^32^ They created a deep learning network to identify such lesions, which had a higher accuracy, sensitivity, and specificity compared to radiologists alone, but an even better result when used in combination with radiologists.^32^ This is particularly relevant, as patients with early esophageal cancers as asymptomatic, and therefore, they are not always referred to endoscopy.^32^ There is a promising future for the potential identification of incidental esophageal cancers using radiomics.

### The use of radiomics in ^18^F-FDG PET/CT scans

^18^F-FDG PET/CT scans play an important role in the management pathway for esophageal cancer, both in the neoadjuvant setting for staging and treatment planning as well as in the adjuvant setting for assessing tumor response to treatment and predicting survival. Radiomics is increasingly being integrated into ^18^F-FDG PET/CT scan interpretation as it offers more detail and consistency than the human eye.

#### Treatment response

##### Textural features

A total of 17 studies were included that addressed the role of radiomics in ^18^F-FDG PET/CT scans to predict treatment response. Standardized uptake values (SUV—SUV_max_, SUV_peak_, and SUV_mean_) in ^18^F-FDG PET/CT are the typical measures used to determine tissue metabolic activity. With the onset of AI, textural analysis biomarkers are being identified to correlate with tumor activity and therefore indirectly, treatment response, and has been suggested to be more sensitive than SUV alone.^33^^, ^^34^ A range of textural features identified in pre-treatment ^18^F-FDG PET/CT have been implicated in predicting tumor response by differentiating responders and non-responders. These include homogeneity,^33^ entropy,^33^ size zone variability,^33^^, ^^35^ intensity variability,^33^^, ^^35^ metabolic tumor volume^35^/tumor volume,^36^ total lesion glycolysis,^35^^, ^^36^ skewness,^37^ inertia,^37^ correlation,^37^ cluster prominence,^37^ run length,^38^ size zone matrix,^38^ short zone high gray emphasis,^38^ and coarseness (as part of a CNN approach).^39^

##### Risk prediction models

Eight of the 17 studies discussed the development of AI-based models to predict tumor response based on textural and/or clinical parameters. Least absolute shrinkage and selection operator (LASSO) logistic regression models have been shown to be good at stratifying patients into high and low risk categories.^40^^, ^^41^ Paul et al. used a feature-based selection model, Genetic Algorithm based on Random Forest which was shown to be superior to other models at risk prediction.^42^ Of note, Van Rossum et al. created a prediction model following their study of 217 patients with EAC, the largest study of its kind, and reported that it was difficult to truly base clinical decision making on such models as associations were often found between textural features and treatment responders, but not to an extent that would confidently separate them from non-responders.^43^ Other studies have shown that risk prediction models that combine textural features alongside histological subtype, Tumor/Node Metastasis (TNM) staging and tumor size can improve the accuracy, specificity and sensitivity.^34^^, ^^44^^, ^^45^ Beukinga et al. showed that the addition of biological tumor markers CD44 and HER2 to their radiomic model also improved prediction of treatment response.^46^

##### Pooled analysis

**Fig. 4:**
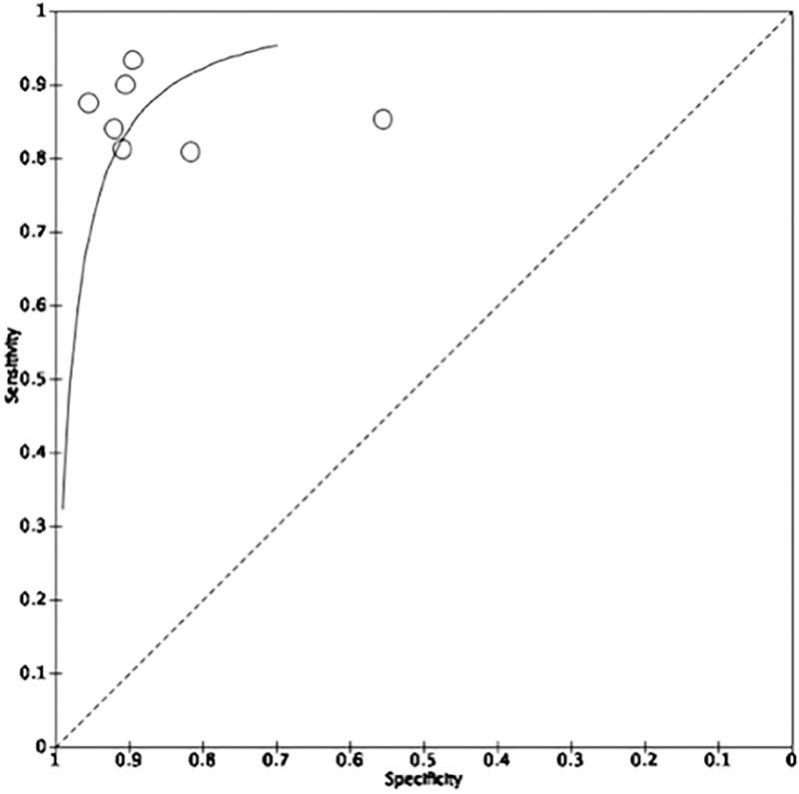
Treatment response in patients with esophageal cancer using radiomics in ^18^F-FDG PET/CT scans.

**Fig. 5:**
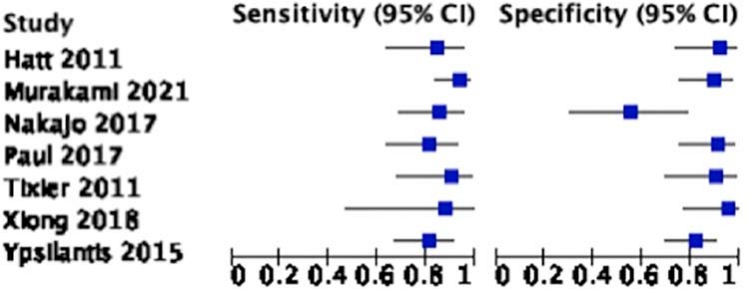
Treatment response in patients with esophageal cancer using radiomics in ^18^F-FDG PET/CT scans.

Seven studies involving 443 patients provided sufficient data of true positive, true negative, false positive and false negative rates for the calculation of sensitivity and specificity. All seven studies assessed the treatment response in patients with esophageal cancer using ^18^F-FDG PET/CT scan scans. The pooled sensitivity and specificity were 86.5% (81.1–90.6) and 87.1% (78.0–92.8), as visualized on the forest plot and summary Receiver Operating Characteristic (ROC) curve (Figs 4 and 5). There was evidence for significant heterogeneity between studies (I^2^ = 72%).

#### Survival

Seven studies focused on the radiomics in ^18^F-FDG PET/CT scans as a means to predict progression-free survival (PFS) and/or overall survival. The reporting in these studies meant that a meta-analysis could not be performed for this section, therefore a narrative review is provided instead.

A single-center study by Karahan Sen et al. evaluating machine learning in textural and metabolic analysis of ^18^F-FDG PET/CT scans in 75 patients noted that total lesion glycolysis and metabolic tumor volume were noted to be higher in the 1- and 5-year non-survivors when compared to survivors at the same time points.^47^ Nakajo et al. noted that ESCC carcinoma patients with a higher metabolic tumor volume, total lesion glycolysis, intensity variability and size zone variability had a shorter PFS and overall survival, however none were an independent factor to strongly influence prognostication.^35^ Paul et al. concurred with Nakajo et al. whereby metabolic tumor volume was deemed to be a good prognostic indicator, in addition to patient factors such as nutritional status and the World Health Organization (WHO) performance status.^42^ Foley et al. developed a prognosis model after studying 403 esophageal cancer patients (of which 237 had EAC).^48^ Foley’s group developed an Automated Decision Tree Learning Algorithm for Advanced Segmentation based prognostic model but reported difficulty in discriminating between the internal and external validation models.^48^^, ^^49^ They observed further radiomic features such as histogram energy, and intensity features such as kurtosis, in addition to total glycolysis volume, as independent features which significantly predict overall survival.^48^^, ^^49^ Xiong et al. specifically studied 30 unresectable esophageal cancers (all ESCC) and noted that wavelet radiomic features were especially helpful at prognostication.^45^

#### Other (histological classification, identification of metastatic disease)

One study discussed the role of radiomics in ^18^F-FDG PET/CT scans to determine histological subtype and noted significant textural differences between EAC and ESCC but with no clear correlative patterns to draw any decisive conclusions.^47^ A further study by Baiocco et al. reported that the lower second order SUV entropy combined with higher second order apparent diffusion coefficient entropy as a good textural feature to identify metastatic disease.^50^

## DISCUSSION

This systematic review included a total of 49 articles, the majority were retrospective single center studies, applying AI technology and radiomics to the diagnosis, treatment response, histological identification, and prognostication of esophageal malignancies. This study suggests the potential value AI has in the modern management of esophageal cancer, with the intent of improving outcomes and individualizing treatment strategies. As all patients with a malignancy will have some form of imaging, the potential breadth of AI assisted technology in radiomics is vast, assisting in reducing inter-observer discrepancies, improving identification of subtle pathology and even out-performing radiologists in certain parameters. More than this, AI in radiomics has proved advantageous in predicting survival outcomes in various stages of disease.

Esophageal cancers have a variable pathological complete response to neoadjuvant treatment ranging from 25% to 50%, therefore it would be beneficial to predict likely treatment outcome prior to initiation of therapy.^23^ Treatment response between the imaging modalities in our study identified that ^18^F-FDG PET/CT imaging had a higher specificity of 87.1% compared to CT scans with 76.1% but sensitivities were comparable between the two modalities at 86.5% and 86.7%, respectively. Rishi et al. achieved the best outcomes in identifying treatment response by using combined CT and PET imaging, achieving an AUC of 0.87.^23^

Lymph node metastases in esophageal malignancy is a highly important prognostic factor, therefore accurate preoperative lymph node identification is vital for decision making. Tan et al. successfully used CT imaging to distinguish lymph node metastasis with an AUC of 0.773 and outperformed size criteria alone.^37^ Wang et al. 2017 also showed improved lymph node identification using SVM as opposed to standard short axis size of the largest lymph node on CT scan.^10^ Lymph node involvement correlates with survival prediction which is of clinical relevance.^10^

Survival prediction in this review has largely been based on combining radiomics and tumor biology and/or TNM staging. Among other studies, Cui et al. successfully produced a machine learning model predicting PFS and overall survival in esophageal cancer patients using a combination of radiomics features and clinical features with the combined models displaying high performance with a C-index of 0.79 for PFS and 0.71 for OS for 3 year outcomes.^28^ Radiomic features provide information about underlying tumor biology and behavior. The use of radiomics in conjunction with patient related factors can predict tumor phenotyping as well as response to treatment and prognosis.

Many of the articles in this study did not report diagnostic sensitivities and specificities of their AI models, as such, a fully comprehensive pooled statistical analysis was not able to be conducted. Due to lacking statistical data, a direct comparison between ^18^F-FDG PET/CT and CT imaging was also not achieved. The paucity of available data may reflect real-world applications of what ^18^F-FDG PET/CT and CT imaging are usually used for. Multiple textural features and radiomic models have been listed in this review to aid in the management of esophageal cancers. Despite ample data, no clear comparisons have been made between the models to allow for accurate analysis in this study. Another identified limitation is that many studies were single centers with a small sample size, especially in the ^18^F-FDG PET/CT cohort. As AI technology is a relatively modern aid to cancer management, this may be expected and highlights the need for larger multicenter trials before accepting AI models into routine management. Many of the studies are also from Asian institutions where ESCC is the predominant histological pathology, as such, the relevance of our findings may need to be interpreted with discretion when applied to an international cohort. Lastly, this paper has not effectively differentiated outcomes based on tumor histology; this may limit application of our findings in the real-life setting.

## CONCLUSION

This meta-analysis has clearly identified key areas of AI use in the management of esophageal malignancies and identified gaps in the current literature. It is evident that in the future machine learning will be integrated into patient management, however, further multicenter trials and comparisons of various radiomics-based models are required prior to implementation.

## Supplementary Material

suppl_doad034

PRISMA_2020_checklist_doad034

Search_strategy_doad034
