# Supplementary material for: Performance of radiomics-based artificial intelligence systems in the diagnosis and prediction of treatment response and survival in esophageal cancer: a systematic review and meta-analysis of diagnostic accuracy
Source: Dis Esophagus. 2023 May 26;36(6):doad034. doi: 10.1093/dote/doad034 (PMC10789236; doi:10.1093/dote/doad034)
Supplement: suppl_doad034 [file suppl_doad034.docx]

| **Author/Year** | **Study design** | **Total (AC, SCC)** | **Timing of imaging** | **AI technology/segmentation** | **Outcome measure** | **Ground truth** | **Sensitivity** | **Specificity** | **AUC** | **Accuracy** |
| --- | --- | --- | --- | --- | --- | --- | --- | --- | --- | --- |
| Wang  2017  DOI:  10.1097/RCT.0000000000000555 | Retrospective | N=131  AC and SCC  (not specified) | Pre and post NACT: platinum based two drug combinations (paclitaxel/cisplatin/nedaplatin) | Support vector machine model  ROI | Identification of lymph node metastases  AI model vs standard CT interpretation  ROC | Histology | - | - | 0.887 |  |
| Takeuchi  2021  DOI:  10.1007/s10388-021-00826-0 | Retrospective | N=457  Test data set of 46 patients  SCC 44  AC 2 | Pre-treatment CT (not specified) | CNN (VGG16) | Identification of oesophageal cancer  CNN vs 2xradiologists | Histology | 0.717 | 0.900 |  | 0.842 |
| Kawahara 2021  DOI: 10.1259/bjr.20210525 | Retrospective | N=104  (all SCC) | Prior to radiotherapy treatment | 13 key radiomic features identified  Model built on least absolute shrinkage and LASSO logistic regression | To predict histological differentiation  AUROC | Histology | 80% | 88.6% | AUC 0.92 | 85.4 |
| Tan 2019  DOI: 10.1007/s00330-018-5581-1 | Retrospective | N=230  All SCC | Pre-treatment CT (arterial phase) | 1576 radiomic features. LASSO logistic regression.  VOI | Model to predict lymph node metastasis  AUROC | Histology |  |  | AUC 0.758 (training set)  AUC 0.773 (test set) |  |

| **Author/Year** | **Study design** | **Total (AC, SCC)** | **Timing of imaging** | **AI technology/segmentation** | **Outcome measure** | **Ground truth** | **Sensitivity** | **Specificity** | **AUC** | **Results** |
| --- | --- | --- | --- | --- | --- | --- | --- | --- | --- | --- |
| Jin 2022  DOI: 10.3389/fonc.2022.892171 | Retrospective | N-215  (not specified) | Pre radiotherapy CT (not specified) | 3D V-Net  2D U-Net  VUMix-Net (CNN) | Radiotherapy planning to assess clinical target volume and gross tumour volume | Histology | - | - | - | AI model better at predicting gross tumour volume and clinical target volume |
| Cao 2022  DOI:  10.1177/15330338211034284 | Retrospective | N=91 (not specified) | Pre radiotherapy (post surgery) | Deep dilated convolutional U network  Clinical target volume auto-segmentation | Radiotherapy planning to assess clinical target volume | Histology | - | - | - | AI model mean Cohen kappa coefficient 0.863  Time for segmentation for clinical target volume was 25 seconds per patient |

| **Author/Year** | **Study design** | **Total (AC, SCC)** | **Timing of imaging** | **AI technology/segmentation** | **Outcome measure** | **Ground truth** | **Sensitivity** | **Specificity** | **AUC** | **Accuracy** | **NPV, PPV** |
| --- | --- | --- | --- | --- | --- | --- | --- | --- | --- | --- | --- |
| Hu  2020  DOI:  10.1001/jamanetworkopen.2020.15927 | Retrospective | n-231  All SCC | Pre treatment - neo-adjuvant chemotherapy followed by surgery | AUROC  Intratumoural and peritumoural features  ROI | Pathological response | Histology | 90.3% | 79.5% | 0.852 (CI 0.753-0.951) | Accuracy 84.3 |  |
| Hu 2021  DOI:  10.1016/j.radonc.2020.09.014 | Retrospective | N=231  Training cohort (n-161)  External testing cohort (n=70)  All SCC | Pre treatment neo-adjuvant chemotherapy followed by surgery | 6 pre trained CNNs  AUROC  ROI | Response to nCRT | Histology | 83.9% | 71.8% | 0.805 (CI 0.696-0.913) | Accuracy: 77.1% (65.6-86.3%) | PPV 70.3%  NPV 84.8%. |
| Jin  2019  DOI:  10.1007/s00330-019-06193-w | Retrospective | N=94 | Pre and post chemoradiotherapy  40-70Gy + cisplatin/5- fluorouracil (5-FU) | 42 radiomic features and 18 dosimetric features  Gross tumour volume | Support Vector Classification (SVM)  Extreme Gradient Boosting algorithm (XGBoost) | Histology | - | - | AUC 0.54 | Accuracy 0.708 |  |
| Rishi  2020  DOI:  10.1111/1754-9485.13128 | Retrospective | N=68  (not specified) | Pre neoadjuvant chemoradiotherapy: 45-56 Gy, cisplatin+5FU | 126 features  -intensity (27 features)  -shape (11 features)  -GLCM (40 features)  -NGTDM (11 features)  -Fractal dimension (8 features)  VOI | Pathological complete response | Histology |  |  | 0.87 | Accuracy 0.77 | TP rate 0.77  FP rate 0.24 |
| Wang 2021  DOI:  10.1088/1361-6560/ac1020 | Retrospective | N=153  All SCC | Pre-treatment CT (esophagectomy, other treatment not specified) | ROI | Survival model based on AI interpretation of histopathology and CT | Histology |  |  | C index in regularised Cox model = 0.694  (CT alone 0.634, histopathology alone 0.664) |  |  |
| Wang 2022  DOI: 10.1155/2022/4034404 | Retrospective | N=154  (training set 116, validation set 38), SCC/AC not specified | Pre-treatment CT (not specified) | Combination of deep learning and handcrafted features (DLR)  ROI | AUC  Kaplan Meier curves | Histology |  |  | AUC 0.984 (primary dataset)  AUC 0.942 (validation dataset) |  |  |
| Li 2021  DOI: 10.1016/j.ijrobp.2021.06.033 | Prospective | N=306 (all SCC) | Pre-treatment CT | 3D-DLRM  ROI | Predict treatment response to CRT  AUROC | Histology |  |  | AUC 0.897 (CI 0.840-0.959) in training cohort  AUC 0.833 (CI 0.654-1) in validation cohort |  | PPV 100 % |
| Riyahi 2018  DOI: 10.1088/1361-6560/aacd22 | Retrospective | N=20 | Pre chemoradiotherapy | Jacobian map, SVM, LASSO  (insight segmentation and registration toolkit) | AUROC  Treatment response | Histology | 94.4% | 91.8% | AUC 0.94 |  |  |
| Hou 2017  DOI:  10.18632/oncotarget.22304 | Retrospective | N=49 | Pre treatment: chemoradiotherapy | 214 radiomic features extracted  SVM (support vector model) and ANN (artificial neural network) model  ROI | Treatment response | Histology | 56.25% | 84.8% | AUC range 0.686-0.727 *for 5 key CT features) |  |  |
| Yip 2014  DOI: 10.1148/radiol.13122869 | Retrospective | N=36 (do not have access to paper?) | Pre and post chemoradiotherapy | Whole tumour texture, entropy, uniformity, mean gray level intensity, kurtosis, standard deviation of histogram, skewness for fine to coarse textures  ROI | Survival  Kaplan Meier  Cox proportional hazards model | Histology |  |  | AUC 0.767  AUC 0.802 |  |  |
| Luo 2020  DOI:  10.1186/s13014-020-01692-3 | Retrospective | N=226 | Pre chemoradiotherapy treatment CT | 7 radiomic features  VOI | LASSO analysis  AUROC  Model to predict complete response of CRT | Histology |  |  | AUC  0.844 training set  0.807 validation set |  |  |
| Qiu 2020  DOI:  10.3389/fonc.2020.01398 | Retrospective | N=206 (all SCC) | CT before neoadjuvant chemoradiotherapy | 8 radiomic features  Gross tumour volume segmentation | LASSO cox proportional hazards regression  Model to predict recurrence free survival in patients with pathological complete response after nCRT and surgery | Histology |  |  | C index 0.746 – radiomics and clinical nomogram  C index 0.685 – radiomics nomogram alone  0.614 – clinical nomogram alone |  |  |
| Cui 2022  DOI:  10.1186/s13014-022-02186-0 | Retrospective | N=204  (all SCC) | Pre and post treatment CT | 944 radiomic features  6 radiomic features for PFS, 3 radiomic features for overall survival  VOI/gross tumour volume segmentation | AUROC  Model for predicting progression free survival and overall survival | Histology | - | - | PFS:  AUC 0.664 (training)  AUC 0.856 (test)  OS:  AUC 0.634 (training)  AUC 0.742 (test) |  |  |
| Yang 2019  DOI: 10.1093/jrr/rrz027 | Retrospective | N=55  (all SCC) | CT pre neo-adjuvant chemoradiotherapy | Radiomic features selected by LASSO  Gross tumour volume | AUROC  Model to predict complete pathological complete response after NACRT | Histology |  |  | AUC 0.84-0.86 (training)  AUC -.71=0.79 (test) |  |  |
| Larue  2017  DOI:  10.1080/0284186X.2018.1486039 | Retrospective | N=165  AC 132  SC 33 | CT pre chemoradiotherapy | 33 textural features  ROI | Random forest model to predict overall survival | Histology |  |  | AUC 0.69 (RF model) |  |  |
| Piazzese 2019  DOI:  [10.1371/journal.pone.0225550](https://doi.org/10.1371/journal.pone.0225550) | Retrospective | N=213 |  | 238 – 2D features and 3D features  4 key radiomic features identified: inverse variance GLCM, large distance emphasis GLDZM, zone distance non uniformity norm GLDZM, zone distance variance GLDZM  2D/3D segmentation | Prognostic model independent of contrast administration | Histology | - | - | - |  |  |

| **Author/Year** | **Study design** | **Total (AC, SCC)** | **Timing of imaging** | **AI technology/segmentation** | **Outcome measure** | **Ground truth** | **Sens** | **Spec** | **PPV/NPV** | **AUC** | **Accuracy** |
| --- | --- | --- | --- | --- | --- | --- | --- | --- | --- | --- | --- |
| Karahan Sen  2021  DOI:  10.1007/s12149-021-01638-z | Retrospective | N=75  AC=13  SCC=62 | Pre-treatment (not specified) | LIFEx software  44 features  Conventional  Histogram based  Shape based  GLCM  GLRLM NGLDM  GLZLM  ROI | To determine histopathological subtype | Histology |  |  |  | AUC 0.780 (CI 0.651-0.910, p 0.002) |  |
| Baiocco  2019  DOI:  [10.1007/s00259-019-04306-7](https://doi.org/10.1007/s00259-019-04306-7) | Retrospective | n-28 | Pre-treatment (staging) | PET MRI  Whole tumour volume segmentation | To identify metastatic disease | Histology | Sens 80% | Spec 80% | PPV 60%  NPV 53% |  | 55% |

| **Author/Year** | **Study design** | **Total (AC, SCC)** | **Timing of imaging** | **AI technology/segmentation** | **Outcome measure** | **Ground truth** | **Sensitivity** | **Specificity** | **AUC** | **PPV/NPV** | **ACCURACY** |
| --- | --- | --- | --- | --- | --- | --- | --- | --- | --- | --- | --- |
| Nakajo  2017  DOI:  10.1007/s00259-016-3506-2 | Retrospective | n=52  SCC only | Pre chemoradiotherapy (CRT): 41-70 Gy with cisplatin.5-fluorouracil | 6 textural heterogeneity parameters:  Entropy, homogeneity, dissimilarity, intensity variability, size zone variability, zone percentage  VOI | Survival prognosis – univariate and multivariate Cox regression analysis:  Progression-free survival (PFS)  Overall survival (OS) | Endoscopy, histology |  |  |  |  |  |
| Paul  2017  DOI:  10.1016/j.compmedimag.2016.12.002 | Retrospective | n=65  SCC=57  AC=8 | Pre CRT: 50 Gy with platinum chemotherapy and 5-fluorouracil | GARF – Genetic Algorithm based on Random Forest (feature based selection model)  19 first order statistics  26 textural features including GLCB, GLSZM, GDLM | GARF compared with other models for prediction of treatment response | Histology | Sens 88% +/- 15 | Spec 72% +/1 23 | AUC 0.750 +/- 0.108 |  |  |
| Karahan Sen  2021  DOI:  10.1007/s12149-021-01638-z | Retrospective | N=75  AC=13  SCC=62 | Pre-treatment (not specified) | LIFEx software  44 features  Conventional  Histogram based  Shape based  GLCM  GLRLM NGLDM  GLZLM  ROI | Overall survival at 1 year and 5 years | Histology, overall survival data | 5 features had significant relationship between 1 year survivors and non survivors  J48:  1 year survival-  AUC 0.581, accuracy 64.29%  5 year survival-  AUC 0.504, accuracy 76.27% |  |  |  |  |
| Foley  2018  DOI:  10.1007/s00330-017-4973-y | Retrospective | N=403  AC=316  SCC=87 | Pre-treatment (surgery alone, NACT, nCRT, definitive chemoradiotherapy, palliative therapy): regimens not specified | ATLAAS tool (Automatic Decision Tree Learning Algorithm for Advanced Segmentation)  **16 texture metrics**  First order histogram metrics: standard deviation, entropy, energy, skewness, kurtosis  GLCMs: homogeneity, entropy, dissimilarity  Coarseness  GLSZMs  Intensity variability  Large area emphasis  Zone percentage | Overall survival | Survival data |  |  |  |  |  |
| Xiong  2018  DOI:  10.1038/s41598-018-28243-x | Retrospective | N=30  All SCC | Pre and mid definitive CRT (unresectable): 20 fractions of radiation doses  Cisplatin and 5-fluorouracil | 440 features in total  14 first order statistics  8 shape and size based features  34 textural features  384 wavelet features  VOI | To create a predictive model for local disease control and PFS  Kaplan-Meier curves | Histology, survival |  |  |  |  |  |
| Karahan Sen  2021  DOI:  10.1007/s12149-021-01638-z | Retrospective | N=75  AC=13  SCC=62 | Pre-treatment (not specified) | LIFEx software  44 features  Conventional  Histogram based  Shape based  GLCM  GLRLM NGLDM  GLZLM  ROI | Overall survival at 1 year and 5 years  5 features had significant relationship between 1 year survivors and non survivors | Histology, survival |  |  | J48:  1 year survival-  AUC 0.581  5 year survival-  AUC 0.504 |  | J48:  1 year survival-  accuracy 64.29%  5 year survival-  accuracy 76.27% |
| Foley 2019  DOI: 10.1016/j.radonc.2018.10.033 | Retrospective | N=449  AC 355  SC 94 | Not specified | ATLAAS segmentation | Creation of a prognostic model | Histology, survival | - |  |  |  |  |

| **Author/Year** | **Study design** | **Total (AC, SCC)** | **Timing of imaging** | **AI technology/segmentation** | **Outcome measure** | **Ground truth** | **Sensitivity (sens)** | **Specificity (spec)** | **AUC** | **PPV/NPV** | **ACCURACY** |
| --- | --- | --- | --- | --- | --- | --- | --- | --- | --- | --- | --- |
| Tixier  2011  DOI: 10.2967/jnumed.110.082404 | Retrospective | n=41  SCC=31 AC=10 | Pre chemoradiotherapy: 60Gy with cisplatin on carboplatin/fluorouracil | AUC ROC  38 textural features based on: intensity histogram, voxel alignment matrix, intensity-size-zone matrix, cooccurrence matrices, neighbourhood intensity-difference matrix | NR vs PR vs CR | RECIST criteria for tumour response | **Non-responders vs partial responders + complete responders:**  **Highest sens = 88%**  SUVmax-  Sens: 53%  SUV mean-  Sens: 71%  SUVpeak:  Sens: 56%  Local homogeneity-  Sens: 88%  Local entropy-  Sens: 79%  Size zone-  Sens: 76%  Intensity variability-  Sens: 85%  **Non-responders + partial responders vs complete responders:**  **Highest sens 92%**  SUVmax-  Sens: 46%  SUV mean-  Sens: 62%  SUVpeak:  Sens: 62%  Local homogeneity-  Sens: 92%  Local entropy-  Sens: 92%  Size zone-  Sens: 92%  Intensity variability-  Sens: 85% | **Non-responders vs partial responders + complete responders:**  **Highest spec = 91%**  SUVmax-  Spec: 73%  SUV mean-  Spec: 45%  SUVpeak:  Spec: 73%  Local homogeneity-  Spec: 74%  Local entropy-  Spec: 82%  Size zone-  Spec: 91%  Intensity variability-  Spec: 75%  **Non-responders + partial responders vs complete responders:**  **Highest spec 91%**  SUVmax-  Spec: 91%  SUV mean-  Spec: 81%  SUVpeak:  Spec: 81%  Local homogeneity-  Spec: 56%  Local entropy-  Spec: 69%  Size zone-  Spec: 69%  Intensity variability-  Spec: 75% | Highest AUC 0.89 |  |  |
| Beukinga  2017  DOI:  10.2967/jnumed.116.180299 | Retrospective | n=97  AC=88  SCC=9  Training set = 97  External validation = no | Pre chemoradiotherapy:  41.4 Gy with carboplatin/paclitaxel | VOI  88 parameters:  7 clinical parameters, 16 geometry features, glycolytic volume based on tumour volume and SUVmean, 19 first order, 24 second-order, 22 higher order textural features  6 different response prediction models constructed using least absolute shrinkage and selection operator regularised logistic regression  VOI | TRG 1 (complete response) vs 2-5 (incomplete response) | Histology | - | - | AUC 0.74 +/- 0.05 |  |  |
| Nakajo  2017  DOI:  10.1007/s00259-016-3506-2 | Retrospective | n=52  SCC only | Pre chemoradiotherapy: 41-70 Gy with cisplatin.5-fluorouracil | 6 textural heterogeneity parameters:  Entropy, homogeneity, dissimilarity, intensity variability, size zone variability, zone percentage  VOI | Treatment response:  Responders vs non-responders (according to RECIST) - AUC | RECIST criteria for recurrence | **Treatment response:**  Intensity variability:  85.3% (29/34)  CI 68.9-95.0  Size zone variability:  94.1% (32/24)  CI 80.3-99.3 | **Treatment response:**  Intensity variability:  55.6% (10/18)  CI 30.8-78.5  Size zone variability:  78% (32/41) |  | **Treatment response:**  **Intensity variability:**  PPV:78.4%(29/37)  CI 61.8-90.2  NPV:  66.7 (10/15)  CI 34.9-90.1  **Size zone variability:**  PPV: 78% (32/41)  CI 62.4-89.4  NPV: 81.8 (9/11)  CI 48.2-97.7 |  |
| Paul  2017  DOI:  10.1016/j.compmedimag.2016.12.002 | Retrospective | n=65  SCC=57  AC=8 | Pre chemoradiotherapy: 50 Gy with platinum chemotherapy and 5-fluorouracil | GARF – Genetic Algorithm based on Random Forest (feature based selection model)  19 first order statistics  26 textural features including GLCB, GLSZM, GDLM | GARF compared with other models for prediction of treatment response | Histology, imaging | 81% +/- 6 | 91 +/- 12 | 0.823 +/- 0.032 |  |  |
| Tan 2013  DOI:  10.1016/j.ijrobp.2012.10.017 | Retrospective | n=20  AC=17  SCC=3 | Pre and post chemoradiotherapy: 50.4 Gy with cisplatin/fluorouracil | 192 features including intensity, texture, geometry, geometry-intensity features  VOI | Pathological response:  TRG 1+2 vs 3-5  AUROC | Histology |  |  | **Highest AUC = 0.80**  Intensity features:  -SUV mean decline  0.79 (p 0.03)  -Skewness 0.76 (p 0.05)  Texture features:  -Inertia 0.85 (p 0.01)  -Correlation 0.80 (p 0.03)  -Cluster prominence 0.78 (p 0.04)  Geometry-intensity feature:  -TGV change 0.74 (p 0.08) |  |  |
| Van Rossum  2016  DOI:  10.2967/jnumed.115.163766 | Retrospective | N=217  AC only | Pre and post chemoradiotherapy: 45 or 50.4 Gy with fluoropyrimidine and either a platinum compound or taxane | 86 features | Multivariable cox analysis  Pathological response:  TRG 1 vs 2-4 | Histology |  |  | 0.77 |  |  |
| Yip  2016  DOI:  10.1088/0031-9155/61/2/906 | Retrospective | N=45  AC=44  SCC=1 | Pre and post chemoradiotherapy: 45-50.4 Gy with cisplatin, 5-fluorouracil, irinotecan/paclitaxel or carboplatin/paclitaxel | GLCM: homogeneity, entropy  RLM: high gray run emphasis, short run high gray run emphasis  GLSZM: high gray zone emphasis, short zone high gray emphasis  ROI | Pathological response (TNM staging)  AUROC | Histology | - | - | Highest AUC = 0.65 +/-0.10 |  |  |
| Beukinga  2018  DOI:  10.1148/radiol.2018172229 | Retrospective | N=70  Ac=65  SCC=8 | Pre and post chemoradiotherapy: 41.4 Gy in 23 fractions with carboplatin/paclitaxel | 113 features | Pathological response  (multivariable logistic regression, least absolute shrinkage and selection operator)  Creating a prediction model | Histology |  |  | 0.81 +/- 0.05 |  |  |
| Xiong  2018  DOI:  10.1038/s41598-018-28243-x | Retrospective | N=30  All SCC | Pre and mid definitive chemoradiotherapy (unresectable): 20 fractions of radiation doses  Cisplatin and 5-fluorouracil | 440 features in total  14 first order statistics  8 shape and size based features  34 textural features  384 wavelet features  VOI | To create a predictive model for local disease control | Histology | Sens 85.7% | Spec 95.7% | **Pre CRT**  **Highest AUC = 0.77**  Correlation LHL  0.76  P0.02  Skewness  0.77  P 0.04  **Mid CRT:**  **Highest AUC 0.83**  Correlation LHL 0.76  P 0.02  Median HLL  0.83  P 0.03  Correlation HLL  0.75  P 0.04  Cluster prominence HLL  0.75  P 0.04 |  | Accuracy 93.3% |
| Ypsilantis  2015  DOI:  10.1371/journal.pone.0137036 | Retrospective | N=107  AC=86  SCC=20  Undefined=1 | Pre-treatment (not specified) | 58 textural features including  Gray level co-occurrence, gray level run length, gray level size zone, gray level difference, fractal based features, neighbourhood gray tone difference  ROI | TRG (Mandard) – pathological response  Comparing two radiomic approaches hand engineered vs CNN | Histology | Sens 80.7% +/- 11.5 | Spec 81.6% +/- 9.2 |  |  | Accuracy 73.4% +/- 5.3 |
| Hatt 2011  DOI:  10.1007/s00259-011-1834-9 | Retrospective | N=50  AC=14  SCC=36 | Pre neoadjuvant chemoradiotherapy: 60 Gy+cisplatin/fluorouracil | Pre MATV, pre entropy, pre homogeneity, pre dissimilarity, pre intensity variability, pre zone percentage  ROI | NR+PR VS CR  Clinical response  RECIST  ROC | Histology/radiological response (RECIST) | **NR+PR VS CR**  **Highest sens 83.3%**  TL:  TA1- 83.3%  TA2- 83.3  FLAB 83.3  TV:  TA1-75  TA2-83.3  FLAB-75  TLG:  TA1-66.7  TA2-75  FLAB-75  **NR VS PR+CR**  **Highest sens 94.6%**  TL:  TA1- 59.5  TA2- 75.7  FLAB 59.5  TV:  TA1-78.4  TA2-94.6  FLAB- 75.7  TLG:  TA1- 92.3  TA2- 69.2  FLAB- 84.6 | **NR+PR VS CR**  **Highest spec 92.1%**  TL:  TA1- 79%  TA2- 73.3  FLAB 65.8  TV:  TA1-81.6  TA2-57.9  FLAB-79  TLG:  TA1-92.1  TA2-86.8  FLAB-92.1  **NR VS PR+CR**  **Highest spec 92.3%**  TL:  TA1- 92.3  TA2- 69.2  FLAB 92.3  TV:  TA1-69.2  TA2-53.9  FLAB-76.9  TLG:  TA1-56.8  TA2-81.8  FLAB-75,7 | **NR+PR VS CR**  **Highest AUC 0.85 (0.73-0.98)**  TL:  TA1- 0.81 (CI 0.65-0.97)  TA2- 0.79 (0.63-0.96)  FLAB 0.79 (0.64-0.94)  TV:  TA1-0.79 (0.65-0.89)  TA2-0.74 (0.59-0.85)  FLAB-0.78 (0.64-0.88)  TLG:  TA1-0.81 (0.62-1.00)  TA2-0.80 (0.61-0.99)  FLAB-0.85 (0.73-0.98)  **NR VS PR+CR**  **Highest AUC 0.86 (0.75-0.98)**  TL:  TA1- 0.78 (0.63-0.93)  TA2- 0.75 (0.61-0.90)  FLAB-0.82(0.70-0.94)  TV:  TA1-0.79 (0.65-0.93)  TA2-0.81 (0.67-0.95)  FLAB-0.84 (0.72-0.96)  TLG:  TA1-0.78 (0.65-0.92)  TA2-0.80 (0.67-0.93)  FLAB-0.86 (0.75-0.98) |  |  |
| Cao 2020  DOI: 10.1016/j.radonc.2020.01.027 | Retrospective | N=159  (SCC only) | Pre-treatment PET: chemoradiotherapy | 944 radiomics features identified, differentially expressed features identified. Least absolute shrinkage and selection operator (LASSO) logistic regression model with 10 fold cross validation – to create a radiomic signature  VOI | AUC  Kaplan Meier analysis  To predict treatment response | Histology, treatment response as per RECIST criteria |  |  | AUC 0.844 (training set)  AUC 0.835 (validation set) |  |  |
| Beukinga 2021  DOI: 10.1007/s00330-020-07439-8 | Retrospective | N=96  (AC=88SCC=8) | Pre neoadjuvant chemoradiotherapy | 3 clinical-radiomic PET features | To assess addition of HER2 and CD44 related biological tumour marker to radiomic models to predict complete response to neo-adjuvant chemoradiotherapy | Histology |  |  | **Highest AUC 0.857**  For the three models:  0.759  0.857  0.816  (for the three models) |  |  |
| Yip 2016  DOI: 10.3389/fonc.2016.00072 | Retrospective | N=54 (not specified AC/SCC) | Pre and post chemoradiotherapy | Textural features | AUC  Kaplan Meier | Histology |  |  | **Entropy** distinguished non responders from complete and partial  Complete AUC 0.79  Partial AUC 0.71  **Change in run length and size zone matrices**  to distinguish partial and complete responders  AUC 0.71-0.76 |  |  |
| Simoni 2020  DOI:  10.3389/fonc.2020.599907 | Retrospective | N=54 (AC 35, SCC 19) | Before induction chemo, after induction chemo, before surgery  (induction chemo was followed by neoadjuvant chemoradiotherapy) | VOI | Pathological response | Histology |  |  | **Highest AUC 0.62-0.74**  SUV max  0.44-0.65  SUV mean  0.43-0.67  MTV  0.62-0.74  TLG  0.47-0.69 |  |  |
| Zhang 2019  DOI: 10.1093/dote/doy096 | Retrospective | N=36  AC 25, SCC 11 | CT pre and post neoadjuvant treatment | Textural features: entropy, correlation, homogeneity, inertia  Segmentation by tumour volume | Pathological response | Histology |  |  |  |  |  |
| Murakami 2021  DOI: 10.3390/diagnostics11061049 | Retrospective | N=98 |  | 4250 radiomic features  LASSO  Gross tumour volume and clinical target volume segmentation | Model for predicting local response to neoadjuvant chemoradiotherapy | Histology | Sens 92.7% | Spec 89.5% | AUC 0.95 |  | 89.6% |
