# Supplementary material for: Performance of radiomics-based artificial intelligence systems in the diagnosis and prediction of treatment response and survival in esophageal cancer: a systematic review and meta-analysis of diagnostic accuracy
Source: Dis Esophagus. 2023 May 26;36(6):doad034. doi: 10.1093/dote/doad034 (PMC10789236; doi:10.1093/dote/doad034)
Supplement: Search_strategy_doad034 [file search_strategy_doad034.docx]

**Search strategy**

(artificial intelligence) OR (radiomics) AND ((esophageal cancer) OR (oesophageal cancer))

(artificial intelligence) OR (machine learning) AND ((esophageal cancer) OR (oesophageal cancer))

(radiomics) AND ((esophageal cancer) OR (oesophageal cancer))
